# Peer Review of “Improved Alzheimer Disease Diagnosis With a Machine Learning Approach and Neuroimaging: Case Study Development”

**DOI:** 10.2196/73454

**Published:** 2025-04-21

**Authors:** Masoud Khani

**Affiliations:** 1 University of Wisconsin-Milwaukee, Milwaukee, WI, United States

**Keywords:** Alzheimer disease, computer-aided diagnosis system, machine learning, principal component analysis, linear discriminant analysis, t-distributed stochastic neighbor embedding, feedforward neural network, vision transformer architecture, support vector machines, magnetic resonance imaging, positron emission tomography imaging, Open Access Series of Imaging Studies, Alzheimer's Disease Neuroimaging Initiative, OASIS, ADNI

*This is a peer-review report for “Improved Alzheimer Disease Diagnosis With a Machine Learning Approach and Neuroimaging: Case Study Development.”*

## Round 1 Review

### General Comments

This paper [1] explores the use of principal component analysis (PCA) and machine learning approaches for the diagnosis of Alzheimer disease (AD) using magnetic resonance imaging and positron emission tomography images from the Open Access Series of Imaging Studies database. The authors propose a system that combines PCA for feature extraction with artificial neural networks (ANNs) and support vector machines (SVMs) for classification. The paper is well structured and presents a clear methodology, but there are several areas where improvements are needed to enhance the rigor and impact of the research.

### Specific Comments

#### Major Comments

1. Methodology justification: The choice of PCA as the sole feature extraction method needs further justification. While PCA effectively reduces dimensionality, it might not capture the most discriminative features of AD. Comparing PCA with other dimensionality reduction techniques like linear discriminant analysis or t-distributed stochastic neighbor emulation could provide a more comprehensive understanding of its effectiveness.
2. Evaluation metrics: The paper primarily focuses on accuracy as the evaluation metric. For medical diagnosis systems, metrics like sensitivity, specificity, precision, recall, and *F*_1_-score are crucial as they provide a better understanding of the model’s performance, especially in imbalanced datasets. Including these metrics would strengthen the evaluation section.
3. Dataset and preprocessing: The preprocessing steps are briefly mentioned but lack detailed explanation. Specific steps for noise reduction, intensity normalization, and any augmentation techniques used should be clearly described. Additionally, the impact of these preprocessing steps on the model’s performance should be discussed.
4. Comparison with existing methods: The paper lacks a thorough comparison with existing state-of-the-art methods. Including a detailed comparison with recent literature, both in terms of methodology and performance, would provide better context and highlight the novelty and effectiveness of the proposed approach.

#### Minor Comments

1. Introduction section: The Introduction provides a good overview of AD and the need for early diagnosis. However, it could benefit from a more detailed discussion of the current challenges in AD diagnosis and how the proposed method aims to address these challenges.
2. Figure and table clarity: Figures and tables should be more clearly labeled and described. For example, in Table 1, it is unclear what “Total cost (Validation)” refers to. Additionally, the axes and legends in figures should be more descriptive to enhance readability.
3. Algorithm parameters: The specific parameters used for the SVMs and ANNs (eg, kernel type for SVMs, number of layers, and neurons for ANNs) should be explicitly mentioned. This would help in reproducing the results and understanding the model configuration.
4. Conclusion and future work: The conclusion should be concise and focus on key findings. The Future Work section could be expanded to include more specific directions for further research, such as exploring different feature extraction methods, incorporating longitudinal data, or integrating other imaging modalities.
5. References: Ensure all references are up-to-date and relevant. Given the rapid advancements in machine learning and medical imaging, some references are slightly outdated. Including more recent studies would enhance the credibility and relevance of the paper.

## Round 2 Review

### General Comments

Thank you for addressing my comments from the previous round of reviews. I appreciate the effort you have put into revising the manuscript. The updated version effectively resolves all the issues I raised, and the manuscript is now clear, well-structured, and scientifically sound.

## Abbreviations

AD: Alzheimer disease
ANN: artificial neural network
PCA: principal component analysis
SVM: support vector machine
